# An association study of *SERPINA1* gene polymorphisms with the risk of metabolic dysfunction-associated steatotic liver disease In an Iranian population: A preliminary case-control study

**DOI:** 10.1016/j.bbrep.2025.101974

**Published:** 2025-03-18

**Authors:** Samira Abdollahi, Abbas Sahebghadam Lotfi, Ramin Saravani, Hamed Taheri

**Affiliations:** a Department of Clinical Biochemistry, Faculty of Medical Science, Tarbiat Modares University, Tehran, Iran; b Cellular and Molecular Research Center, Research Institute of Cellular and Molecular Sciences in Infectious Diseases, Zahedan University of Medical Sciences, Zahedan, Iran; c Department of Clinical Biochemistry, Zahedan University of Medical Sciences, Zahedan, Iran; d Department of Internal Medicine, Ali-Ibn-Abitaleb Hospital, Zahedan University of Medical Sciences, Zahedan, Iran

**Keywords:** MASLD, *SERPINA1*, A1AT, Single nucleotide polymorphisms (SNPs)

## Abstract

**Background:**

Metabolic dysfunction-associated steatotic liver disease (MASLD) is a type of fat accumulation in the liver that can lead to cirrhosis and chronic liver disease. MASLD is recognized as the most frequent of liver-associated deaths worldwide. The *SERPINA1* gene encodes a serine protease protein that plays a pivotal role in the pathogenesis of liver deficiencies. In this study, we aimed to evaluate the genetic association between rs6647 (M1), rs709932 (M2), and rs1303 (M3) variants in the *SERPINA1* gene and the risk of MASLD in an Iranian population.

**Methods:**

In this case-control study, 120 patients affected by MASLD and 120 healthy subjects participated. The Nephelometry system measured serum levels of α1-antitrypsin (A1AT). Biochemical tests were conducted to assess serum levels of blood parameters using commercially available kits. DNA extraction was performed using the salting-out method, followed by the amplification refractory mutation system-polymerase chain reaction (ARMS-PCR) method for genotyping. Statistical analysis was performed by SPSS v16.0.

**Results:**

The findings showed that the rs6647 G allele significantly increased the risk of MASLD. The G allele in codominant, dominant, and over-dominant models caused an increase in the risk of MASLD. Additionally, the rs709932 T allele was more frequent among patients compared to healthy subjects and significantly enhanced the risk of MASLD. The T allele in the codominant and recessive models indicated a high risk for MASLD in our population. The G allele of rs1303 caused an enhancement in the mean serum levels of A1AT in the MASLD group.

**Conclusions:**

Our results show an association between *SERPINA1* gene variants and the risk of MASLD. The rs6647 (M1) and rs709932 (M2) variants of the *SERPINA1* gene increased the risk of disorder in our population.

## Highlights

• rs6647 G allele increased the risk of MASLD, statistically.
• rs709932 T allele significantly enhanced the risk of MASLD.
• The G allele of rs1303 caused an enhancement in the mean serum level of A1AT in MASLD group.
• results showed an association between SERPINA1 gene variants and the risk of MASLD.

## 1 Introduction

**Fig. 1:**
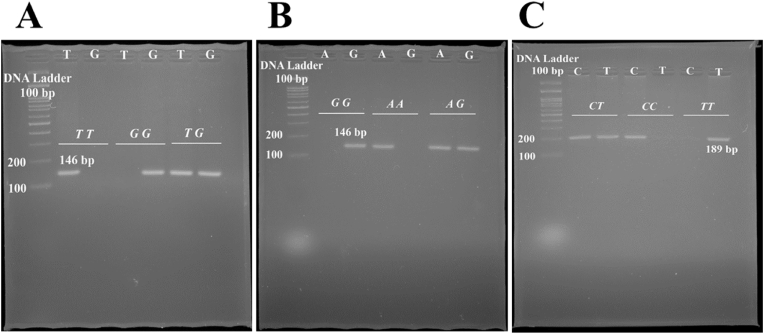
Gel photograph of PCR amplification products of the *SERPINA1* rs1303 T/G (A), rs6647 A/G (B), rs709932 C/T (C) polymorphisms.

Metabolic dysfunction-associated steatotic liver disease (MASLD) is a common liver disease that has become prevalent in recent years, characterized by the accumulation of fat in the form of triacylglycerol. The main cause of fat over-accumulation is a defect in the underlying mechanisms of fat synthesis in the liver [1,2]. In these mechanisms, other causes of fat accumulation, including alcohol consumption, different types of hepatitis, and drugs that induce steatosis, do not participate [3]. This situation is often referred to as cardiometabolic disorder because MASLD is associated with several comorbidities including type 2 diabetes mellitus (T2DM), hypertension, and dyslipidemia [4]. The manifestation of MASLD can vary from a simple accumulation of fat with no symptoms, resembling a metabolic state, to symptomatic non-alcoholic steatohepatitis (NASH). MASLD is symptomatic in most affected individuals and is linked to metabolic features, including obesity [5]. In contrast, NASH is more aggressive in most patients and is associated with balloon degeneration, hepatic lobular inflammation, and fibrosis [6]. To replace the damaged cells, stellate cells produce type I collagen to compensate for hepatocytes, resulting in NASH progression followed by fibrosis, cirrhosis, and their clinical outcomes [7] (see Fig. 1).

The mean estimated prevalence of MASLD globally is about 24 %. The lowest frequency of MASLD has been recorded in the African population, while the highest prevalence is found in South America and Asia, at 30 % and 32 %, respectively [4].

The document illustrates a complex interaction of various factors, including environmental and acquired factors, genetic factors, and metabolic factors, which contribute to the occurrence and etiology of MASLD. Current findings indicate that MASLD involves extra accumulation of fatty acids due to an imbalance in the uptake and intake of lipid metabolism caused by alterations in lifestyle, sedentary behavior, and metabolic syndromes. High-energy diets and hepatic lipogenesis led to an increase in fatty acid input, while the oxidation of fatty acids remains insufficient to mitigate the increased levels of fatty acids. Moreover, inadequate dietary habits among individuals with MASLD could result in alterations to the microbiota, linked to reduced production of short-chain fatty acids (SCFAs), elevated intestinal permeability, and the translocation of bacteria or their byproducts from the gut to the liver, triggering inflammatory responses. consequently, the activation of stellate cells and the release of collagen participate in fibrosis and MASLD progression [8]. Family studies describe an elevated risk for MASLD among relatives of affected individuals, predicting a genetic predisposition to MASLD [9,10]. Additionally, twin studies declare that genetic predisposition contributes to susceptibility to MASLD and the development of cirrhosis and fibrosis [11]. Furthermore, the different prevalence of MASLD among different ethnicities is likely to derive from the genetic background of these populations. It is shown that people in some populations respond to metabolic situations including obesity in a different process by declining steatosis and hypertriglyceridemia. The results of the GWAS study confirmed that these differences can be explained by the genetic content of individuals [12]. In recent years several GWAS and gene-candidate studies have been performed on the MASLD that construct our findings of this disorder. These studies confirmed the association of genetic variants of several genes including *PNPLA3*, *TM6SF2*, and *HSD17B13* as well as some non-coding genes including *miR-122* and *miR-34* with the incidence of MASLD [13].

The *Serpin Family A member 1 (SERPINA1)* gene located on chromosome 14q32.13 encodes 7 exons. This gene provides instructions for making a protein called alpha-1 antitrypsin (A1AT), which is a type of serine protease inhibitor (serpin). Serpins help control several types of chemical reactions by blocking (inhibiting) the activity of certain enzymes. The destructive effects of neutrophil elastase on the lung tissue are neutralized by A1AT. The liver is the main source of A1AT while other organs including the kidney and intestinal cells synthesize it on a minor level [14]. The 5′-UTR splicing of the A1AT is hypothesized to be responsible for tissue-specific expression of this protein [15]. IL-6 and IL-1 regulate the promoter of *SERPINA1* in response to inflammation. Generally, the documents support that A1AT participates in inflammatory processes through its protease-inhibitory mechanism. In addition, this protein seems involved in tissue repair and regeneration. Chronic inflammation is a crucial stage in cancer development, it is hypothesized that augmented levels of A1AT assist the cancer progression. In this view, several studies confirmed the association of enhanced levels of A1AT with developed cancer steps [16]. Moreover, some documents confirmed the association of genetic variants of *SERPINA1* with liver cirrhosis, inflammation, and fibrosis. In addition, some alleles of *SERPINA1* caused an increased risk for mortality related to the liver.

In the current study, we aimed to evaluate the association of *SERPINA1* rs6647 (M1) (Val213Ala), rs709932 (M2), and rs1303 (M3) variants with levels of A1AT in serum and risk of MASLD in an Iranian population.

## 2 Materials and methods

### 2.1 Participants

In the current case-control study, 120 affected by MASLD whose disorder had been diagnosed were included as the case group. The ultrasound (US) findings of these patients confirmed the presence of fatty liver in the absence of alcohol intake. A hepatologist diagnosed the disorder based on the American Association for the Study of Liver Diseases (AASLD) criteria, updated in 2023 [17]. The 120 healthy age and gender-match individuals who were referred to Ali-ebne-Abitaleb Hospital, Zahedan for routine annual checks were contributed as the control group. The medical consultant confirmed no presence of comorbidities including type 2 diabetes mellitus, metabolic disorders, and cardiovascular diseases. The participants with alcohol consumption, hepatic drug intake, cancers, viral infection, pregnancy, and other genetic disorders related to the liver were excluded from the next steps. All subjects received related information about the current study and filled out the consent form.

### 2.2 Genotyping

About 3 ml of whole blood from all participants was drained into EDTA-containing tubes. The DNA was extracted using a standard salting-out method protocol. A microspectrophotometer (BOECO, Germany) optimized the quantity of all extracted DNA. The mean ratio of OD260/280 of all extracted DNA was in the range of 1.8–2.0. The genotyping method was based on the Amplification refractory mutation system polymerization chain reaction (ARMS-PCR) method. In this method, two primers act as allele-specific primers. Each subject is assigned two tubes containing a type of allele-specific primer. The primer sequences of interested variants in the *SERPINA1* gene were designed by Generunner software. The sequences of primers and related information have been presented in Table 1. Almost, each reaction tube contains 10 μl of 2X premix master mix (Parstous, Iran), 0.6–0.8 μl of appropriate primer (10 p.m./μl), 0.6 μl of extracted DNA, and up to 20 μl of sterilized H_2_O. The Veriti™ 96-well Thermal Cycler ABI appliance was recruited to genotype *SERPINA1* variants. The PCR reaction was started with a pre-denaturation stage at 95 °C for 10 min followed by a 3-step amplification repeated for 30 cycles containing a denaturation step at 95 °C for 30s, annealing at appropriate temperature for 30 (based on Table 1), and extension step at 72 °C for 30s, and final extension stage at 72 °C for 10 min. The PCR products were separated on a 2 % gel agarose. Finally, the PCR bands were visualized by a transluminator system using safe dyes.

**Table 1:** Primer sequences used for genotyping *SERPINA1* polymorphisms.

SNP	Function	Sequence	Genotyping method	Amplified bands (bp)	Annealing Temp.
rs1303 T/G	Missense Variant	F: TTCTTTAATGTCATCCAGGG R (T): ACCCTTTGTCTTCTTAATGATTGGA R (G): ACCCTTTGTCTTCTTAATGATTGGC	ARMS-PCR	T/G: 146	59
rs6647 A/G	Missense Variant	F (A): ATAGGCACCTTCACGGTGGTAA F (G): ATAGGCACCTTCACGGTGGTAG R: CTGCTACACTCTTCCAAACC	ARMS-PCR	A/G: 146	61
rs709932 C/T	Missense Variant	F (C): TGTCTGGCTGGTTGAGGGTTC F (T): TGTCTGGCTGGTTGAGGGTTT R: CCAACAGCACCGATATCTTC	ARMS-PCR	C/T: 189	63

SNP, Single nucleotide polymorphism; bp, Base pair; F, Forward; R, Reverse.

### 2.3 Biochemistry analysis

The serum level of A1AT was measured by the Nephelometry kit based on the manufacturer's instructions. In this method, the antibody-A1AT complex scattered the light and a detector collects this light. This technique quantifies the amount of collected light as a unit of concentration [18]. The blood levels of alanine transaminase (ALT), aspartate aminotransferase (AST), alkaline phosphatase (ALP), and total protein (PT) were calculated by a commercially available kit (Delat Darman company, Iran) according to producer protocol and a biochemistry analyzer machine.

### 2.4 Statistical analysis

The T-student and Mann-Whitney U tests were used for the comparison of quantitative variables between case and control groups. The Chi-square test was utilized for the evaluation of differences in the qualitative variable between studied groups. The logistic regression analysis was recruited for association analysis of different genotypes with MASLD risk through odd ratio (OR) and 95 % confidence interval (CI). The p-value under 0.05 is considered statistically significant. The categorical variables are represented as frequency and percentage while continuous variables are expressed by mean and standard deviation (SD). All statistical analysis was performed by IBM SPSS package v16.0 (SPSS Inc, USA).

## 3 Results

### 3.1 Demographic results

The results of clinical and demographic characteristics confirmed that patient and normal groups were adjusted in terms of gender (Table 2). The body mass index (BMI) in the MASLD patients was higher compared to the healthy group. Specifically, the height of participants was in the same range and the main difference was seen in the weight of subjects. In other words, MASLD patients had more weight than the control group. In addition, subjects in the MASLD group consume high-fat products compared to the normal group. The history of patients showed that the majority of patients had moderate fibrosis considered stage 2 of fibrosis. The biochemical analysis revealed that the amount of AST, ALT, ALP, and TP was significantly increased in the serum of patients with MASLD compared to the reference group.

**Table 2:** Clinical and demographic characteristics of *MASLD* patients and controls.

Parameter evaluated	*MASLD* (N); (mean ± SD)	Controls (N); (mean ± SD)	*p*-value
Gender			
⁃ Male	43	45	0.789
⁃ Female	77	75	
Ethnicity			
⁃ Fars	61	63	0.796
⁃ Baloch	59	57	
Age (*year*)	44.64 ± 11.39	41.46 ± 10.76	**0.025**
Height (*m*)	1.67 ± 0.06	1.68 ± 0.07	0.773
Weight (*kg*)	76.92 ± 14.67	63.36 ± 5.89	**<0.001**
Diets			
⁃ Low-fat	15	116	**<0.001**
⁃ High-fat	105	4	
BMI (*kg/m*^*2*^)	27.61 **±** 5.30	22.53 ± 1.94	**<0.001**

⁃ Underweight or <18.5	0	3	
⁃ Ideal or 18.5–24.9	44	106	
⁃ Overweight or 25-30	38	8	
⁃ Obese or >30	37	1	
Grade			
⁃ Fatty liver (fibrosis stage 0)	0		
⁃ MASH with mild fibrosis (fibrosis stage 0 or 1)	2	–	–
⁃ MASH with moderate fibrosis (fibrosis stage 2)	105		
⁃ MASH with advanced fibrosis (fibrosis stage 3)	12		
⁃ Cirrhosis (fibrosis stage 4)	1		
A1AT (*mg/dl*)	113.73 ± 4.19	105.55 ± 2.54	0.401
AST (*mg/dl*)	40.81 ± 37.01	19.02 ± 11.41	**<0.001**
ALT (*mg/dl*)	38.68 ± 48.49	11.61 ± 17.08	**<0.001**
ALP (*mg/dl*)	212.38 ± 75.76	140.73 ± 47.72	**<0.001**
TP (*mg/dl*)	7.66 ± 0.68	6.99 ± 0.98	**<0.001**

### 3.2 Genotyping findings

Table 3 depicts the results of the genotyping of *SERPINA1* variants in the studied participants. The results of genotyping showed that the studied population was in Hardy-Weinberg equilibrium (HWE). The results of rs6647A > G genotyping revealed that the G allele increased the risk of MASLD, statistically (OR = 1.71, 95%CI = 1.10–2.60, p < 0.028). AG vs. AA genotype increased the risk of MASLD, strongly (OR = 2.44, 95%CI = 1.38–4.18, p = 0.004). In addition, AG + GG compared to the AA genotype caused an enhancement in the risk of disorder by OR = 2.20 (95%CI = 1.29–3.69, p = 0.008). Moreover, the G allele in over dominant model, AG versus AA + GG enhanced the incidence of MASLD compared to the healthy group (OR = 2.41, 95%CI = 1.39–4.14, p = 0.005). The results of genotyping regarding rs709932C > T revealed that the T allele was more frequent in MASLD patients compared to healthy subjects (OR = 1.77, 95%CI = 1.19–2.57, p = 0.010). The T allele in the TT versus CC + CT model had a sharp effect on the incidence of MASLD (OR = 6.12, 95%CI = 2.25–16.45, p < 0.001). Furthermore, the TT genotype compared to the CC genotype caused a statistically significant increase in the risk of MASLD (OR = 5.95, 95%CI = 2.12–16.58, p < 0.001). The findings of genotyping of rs1303T > G showed no statistical differences between the MASLD group and healthy subjects.

**Table 3:** Allelic and genotypic distribution of *SERPINA1* polymorphisms.

SNP	*MASLD*, n (%)	Control, n (%)	Genetic model	OR (95 % CI)*∗*	*p*-value*∗*
rs6647 A/G					
AA	60 (50.0)	82 (68.3)		1 [ reference]	
AG	54 (45.0)	31 (25.8)	Codominant 1	2.44 (1.38–4.18)	**0.004**
GG	6 (5.0)	7 (5.8)	Codominant 2	1.19 (0.39–3.69)	0.794
HWE	0.159	0.095	Dominant	2.20 (1.29–3.69)	**0.008**
			Recessive	0.88 (0.31–2.65)	0.785
			Over dominant	2.41 (1.39–4.15)	**0.005**
A	174 (72.5)	195 (81.3)	Allelic	1 [ reference]	
G	66 (27.5)	45 (18.7)	Allelic	1.71 (1.10–2.60)	**0.028**
rs709932 C/T					
CC	50 (41.7)	59 (50.8)		1 [ reference]	
CT	45 (37.5)	56 (45.0)	Codominant 1	1.01 (0.58–1.65)	0.869
TT	25 (20.8)	5 (4.2)	Codominant 2	5.95 (2.12–16.58)	**<0.001**
HWE	0.018	0.062	Dominant	1.39 (0.82–2.28)	0.251
			Recessive	6.12 (2.25–16.45)	**<0.001**
			Over dominant	0.71 (0.42–1.16)	0.158
C	145 (60.4)	174 (72.5)	Allelic	1 [ reference]	
T	95 (39.6)	66 (27.5)	Allelic	1.77 (1.19–2.57)	**0.010**
rs1303 T/G					
TT	87 (72.5)	76 (63.3)		1 [ reference]	
TG	24 (20.0)	37 (30.8)	Codominant 1	0.59 (0.32–1.06)	0.065
GG	9 (7.5)	7 (5.8)	Codominant 2	1.15 (0.41–3.19)	0.830
HWE	0.001	0.0388	Dominant	0.68 (0.39–1.18)	0.131
			Recessive	1.35 (0.50–3.66)	0.623
			Over dominant	0.59 (0.36–1.03)	0.058
T	198 (82.5)	189 (78.8)	Allelic	1 [ reference]	
G	42 (17.5)	51 (21.2)	Allelic	0.83 (0.51–1.28)	0.305

SNP, Single-nucleotide polymorphism; CI, confidence interval; OR, odds ratio; HWE, Hardy weinberg equilibrium; *p*-value∗ and OR (95%CI)∗, age adjusted. Codominant 1 and Codominant 2 represent the heterozygous and homozygous codominant models, respectively. *p* < 0.05 is considered statistically significant.

### 3.3 The serum level of A1AT among studied genotypes

Table 4 shows the results of the evaluation of A1AT levels in different genotypes of studied variants. In terms of rs1303, the TT genotype was associated with the lower amount of A1AT level while the GG genotype was correlated with the highest level of A1AT. In the case of rs6647, participants with the AG genotype had a minimum mean level of A1AT with 103.94 ± 3.95 while their counterparts with the GG genotype recorded the highest mean level of A1AT with 128.158 ± 16.80. Subjects with CC genotypes of rs709932 had the lowest amount of A1AT compared to TT genotypes.

**Table 4:** The serum level of A1AT in different genotypes of studied *SERPINA1* variants.

SNP	Genotype	A1AT *(mg/dl)*
rs1303		
	TT	108.40 ± 2.75
	TG	110.79 ± 4.61
	GG	120.75 ± 16.67
rs6647		
	AA	111.35 ± 3.03
	AG	103.94 ± 3.95
	GG	128.15 ± 16.80
rs709932		
	CC	103.29 ± 2.88
	CT	110.56 ± 3.56
	TT	129.57 ± 10.97

SNP, Single-nucleotide polymorphism; A1AT: alpha 1 antitrypsin.

### 3.4 The correlation between A1AT levels and genotypes

Table 5 depicts the correlation of the mean level of A1AT with the recessive genetic model of studied variants in MASLD patients and their healthy counterparts. Based on the results, healthy subjects with GG genotype of the rs1303 had a lower amount of A1AT compared to TT + TG genotypes. While MASLD patients with GG genotypes had more high levels of A1AT compared to TT + TG genotypes. In other words, it seems that the G allele in MASLD patients caused an increase in the level of A1AT.

**Table 5:** The correlation of mean serum level of A1AT with recessive genetic model of studied *SERPINA1* variants between MASLD and control group.

SNP	Group	Genotype	A1AT
rs1303	MASLD	TT + TG GG ***p*-value**	110.45 ± 42.68 154.11 ± 65.48 **0.013**
	Control	TT + TG GG ***p*-value**	107.27 ± 26.26 77.86 ± 39.82 **0.036**
rs6647	MASLD	AA + AG GG ***p*-value**	112.45 ± 42.81 138.00 ± 89.12 0.413
	Control	AA + AG GG ***p*-value**	104.67 ± 27.98 119.71 ± 23.28 0.184
rs709932	MASLD	CC + CT TT ***p*-value**	107.76 ± 38.67 136.40 ± 62.49 0.050
	Control	CC + CT TT ***p*-value**	105.99 ± 27.77 95.40 ± 31.43 0.641

SNP, Single-nucleotide polymorphism; A1AT: alpha 1 antitrypsin; *p* < 0.05 is considered statistically significant.

## 4 Discussion

The findings of this study showed that both rs6647A > G and rs709932C > T caused an increase in the risk of MASLD disorder in our studied population. The G allele of rs6647A > G in codominant, dominant, and over-dominant models increased the risk of MASLD, statistically. In addition, the T allele of rs709932C > T in codominant and recessive models enhanced the incidental risk of disorder, significantly. The difference in the A1AT levels between MASLD patients and the reference group was not significant in our population. While the GG genotype of rs1303 caused an increase in the mean levels of A1AT in MASLD subjects.

The *SERPINA1* gene has two distinct promoters: the upper one is related to macrophages, while the lower one is specific to hepatocytes. Each of these two promoters is turned on in the appropriate cells [15]. The main source of A1AT is hepatocyte cells, which release this protein into the blood circulation. The major role of A1AT is to protect alveolar lung cells from non-specific damage caused by neutrophil serine protease including neutrophil elastase (NE), and proteinase-3 [19]. A1AT deficiency (AATD) is an autosomal recessive disorder caused by mutations in the *SERPINA1* gene, leading to diminished levels of A1AT in plasma. To date, more than 100 variants have been identified in the *SERPINA1* gene. They are named based on the migration through the isoelectric field (IEF) system. The earlier the alphabetic letter is, the faster the migration is. The most clinically relevant mutations Pi∗Z (rs28929447) and Pi∗S (rs175880) show very slow and slow migrations on the IEF system, respectively. Meanwhile, the Pi∗M allele displays a medium or normal movement [20]. The classic homozygous of Pi∗Z (Pi∗ZZ) caused a chronically decreased concentration of A1AT in serum up to 80 % and put individuals at high risk for lung disorders [20]. This mutation caused an accumulation of protein in the production sites like in the endoplasmic reticulum. The co-inheritance of Pi∗Z and Pi∗S alleles is related to an intermediate serum level of A1AT and is associated with a moderate risk for lung disorders. The main mechanisms for diminishing the effect of A1AT mutations are protein degradation associated with endoplasmic reticulum and autophagy. It is documented that increased autophagy reduces the accumulation of Z-A1AT and liver injuries [21]. JNK signaling pathway is involved in cell growth, differentiation, tumorigenesis, and cell death in hepatocytes. Moreover, it contributes to inflammation and fibrosis. It has been observed that activation of JNK in the livers of Z-A1AT (ATZ) patients resulted in the activation of *forkhead box O3* (FOXO3) [22,23]. JNK activation via the NF-kB signaling pathway activates FOXO3 followed by upregulation of hsa-miR-34b/c. hsa-miR-34b/c suppressed the profibrotic activity of platelet-derived growth factor resulting in blocking fibrosis. The deletion of has-miR-34b/c in mice models triggers liver fibrosis development [24].

These mutations are categorized as deficient or null. The deficient mutations caused a decrease in the serum levels of A1AT and the null are ones caused by nonsense or frameshift mutations that lead to premature stop codons and are followed by undetectable levels of A1AT [25]. The deficient mutations in *SERPINA1* caused the retention of A1AT in the liver followed by hepatocyte toxicity, and lack of A1AT in blood circulation leads to lung disorders [26]. A1AT is the main antagonist of neutrophil elastase, therefore decreased levels of A1AT lead to damage in lung tissue and respiratory disorders [27]. In addition, some cases of AATD may trigger liver disorders including cirrhosis and hepatocellular carcinoma (HCC).

In line with the study of Malik et al., the results of an exome-wide association study showed that rs6647A > G is associated with an increased risk of large artery atherosclerotic stroke (LAS). The results of the microscale thermophoresis method revealed that the dissociation constant between A1AT and its main target, NE was lower in the presence of the G allele compared to the A allele of rs6647. In other words, plasma components interfere more strongly with the G allele [28]. In a study by Liu Q et al., the association of rs6647A > G with the level of A1AT and risk of LAS was evaluated. The findings revealed that rs6647 plays a role in different human tissues including brain and blood based on the enhancer analysis. The results of expression quantitative trait loci (eQTRs) in the GTEx v.6 datasets showed a probable association between rs6647A > G and expression levels of the *SERPINA1* gene in prostate and adipose-subcutaneous tissue. eQTRs are genetic loci that could alter the expression levels of mRNAs. The study of eQTRs may help uncover the role of genes in specific tissues [29]. The highest correlation between the rs6647 G allele and expression of *SERPINA1* was seen in blood (26). In a study on chronic obstructive pulmonary disease (COPD) patients in the Mexican population, the authors did not report statistically significant differences in frequencies of rs709932 and rs1303 between patients and the healthy groups [30]. To date, the current study is the first one on the evaluate of the association between M1, M2, and M3 variants of the *SERPINA1* gene with the risk of MASLD.

Precision medicine tries to define individuals in subsets based on their genetic background, environmental features, and lifestyle. Precision medicine promotes a deeper concept of disorders to achieve better diagnosis, treatment, and outcome of disorders according to personal characteristics [31]. The recent progression in genetic composition and system biology of MASLD resulted in a series of studies to evaluate the application of personalized medicine in this disorder [32,33]. In the diagnosis stages, besides all biomarkers and imaging systems that are used, the MASLD needs a series of items that define the extension and severity of hepatic involvement. For example, the LDE system is a descriptor of parameters related to the liver (L), determinants of disorder that are related to each patient (D), and extension of hepatic involvement (E) which is a routine of a disease same as MASLD [34]. The importance of genetic variations is uncovered in this cut-off edge era such as precision medicine. The genetic variants could be employed as biomarkers for early-stage diagnosis. In addition, genetic variants could affect the secondary structure and interaction of proteins and impact drug efficiency.

Our study comes with limitations. Our population size is small and may affect the proper frequency of alleles. In addition, we faced some challenges in the laboratory by using more highly advanced techniques. We recommend that the next studies evaluate the probable effect of *SERPINA1* variants on the expression level of this protein employing some techniques including quantitative real-time PCR or RNA seq. In silico prediction impact of these variants on the secondary structure of *SERPINA1* and interaction of A1AT with its counterparts, seem to be considered in next steps.

## 5 Conclusion

Our findings showed that rs6674A > G (M1) and rs709932C > T (M2) variants of the *SERPINA1* gene were associated with the risk of MASLD. These genetic variants increased the risk of MASLD in our population, statistically.

## CRediT authorship contribution statement

**Samira Abdollahi:** Writing – original draft, Methodology, Formal analysis, Data curation, Conceptualization. **Abbas Sahebghadam Lotfi:** Supervision, Project administration. **Ramin Saravani:** Writing – review & editing, Writing – original draft, Supervision. **Hamed Taheri:** Writing – original draft, Validation.

## Ethical approval

The current study was approved by the ethics committee of Tarbiat Modares University Code: IR.MODARES.REC.1402.075.

## Declaration of competing interest

The authors declare that they have no known competing financial interests or personal relationships that could have appeared to influence the work reported in this paper.

## Data Availability

Data will be made available on request.
